# Case Report: Surgical Treatment of High-Flow Coronary Fistulas for the Pulmonary Artery

**DOI:** 10.21470/1678-9741-2018-0327

**Published:** 2020

**Authors:** Thales Cantelle Baggio, Larissa Sebold, Igor Cordeiro de Oliveira

**Affiliations:** 1 Hospital e Maternidade Jaraguá, Jaraguá do Sul, SC, Brazil.; 2 Universidade Regional de Blumenau, Ringgold Standard Institution, Blumenau, SC, Brazil.

**Keywords:** Coronary Artery Disease (Pathology, Pharmacology, Physiology, Etc.), Hemangioma, Coronary Sinus, Coronary Angiography, Pulmonary Artery, Delayed Diagnosis, Drainage

## Abstract

Coronary fistulas are rare anomalies that can affect approximately 1% of the population, presenting few specific symptoms, and are often found occasionally in coronary angiography. Here we describe the case of a 61-year-old patient with complaints of precordialgia and dyspnea since adolescence, with late diagnosis of coronary fistulas with drainage to the pulmonary artery, and with unsuccessful percutaneous treatment.

Therefore, she underwent open surgery for the correction of the already known fistulas, in addition to the hemangioma involving such vessels, which made the understanding and resolution of this case more complex.

**Table:** 

Abbreviations, acronyms & symbols	
CCAAs	= Congenital coronary artery anomalies
CAF	= Coronary artery fistula
ICU	= Intensive care unit

## INTRODUCTION

Congenital coronary artery anomalies (CCAAs) are a rare heterogeneous group with a prevalence of approximately 1% in the general population^[1]^. First described by Galene and Versalius, approximately 2,000 years ago^[2]^, CCAAs comprise changes of origin, structure, and pathway, which can generate benign manifestations without hemodynamic or potentially serious signs, and may cause myocardial ischemia and sudden death^[1,3]^. Most of these anomalies are not perceptible by physical examination, being asymptomatic and requiring a high degree of clinical suspicion^[3]^. The classification of CCAAs is divided in two subgroups: 1) coronary artery anomalies originated from the opposite coronary sinus and 2) abnormalities in which the left coronary artery originates from the pulmonary artery^[4]^. The anomalous involution, the division of the *truncus arteriosus,* or a change in the position of the endothelial buds in the embryonic period can lead to the development of these coronary anomalies^[5,6]^, and the persistence of embryonic sinusoids beyond the expected time is intimately related to the formation of coronary fistulas^[7,8]^. Coronary artery fistula (CAF) is a subtype of anomaly, in which an abnormal vessel originating from a coronary artery circumvents the normal capillary bed and ends within the large vessels or cardiac chambers. In the analysis of cases submitted to invasive coronary angiography, the CAFs seem to have a prevalence ranging from 0.05% to 0.25%^[1,9]^, and approximately 20% of them drain to the right heart chambers and to the pulmonary artery^[10]^. Right CAFs are the most prevalent, occurring in 70% of the cases^[11]^.

Approximately 45% of the patients with CAF are symptomatic and it is present, on physical examination, a continuous murmur near the location of the fistula^[9,12]^. The symptoms, when present, depend on the magnitude of blood flow deviation, most of which are non-specific, such as: fatigue, exertional dyspnea, palpitations, chest pain at rest, and bradycardia^[13]^.

As previously mentioned, this type of coronary fistula is a common manifestation of congenital anomalies^[1,14]^ and is associated with other anomalies, such as: patent ductus arteriosus, tetralogy of Fallot, and ventricular septal defect, setting an incidence between 0.2% to 0.4% among coronary fistulas^[9,15]^. The presentation, age, and nature of the left coronary artery with origin from the pulmonary artery are variable and depend on the existing collateral circulation. This anomaly should be suspected in children with symptoms of heart failure, electrocardiographic changes, and decreased left ventricular systolic function^[3]^.

In addition to this, coronary-pulmonary fistula can be caused by iatrogenesis, and thus observed after performing procedures such as: myocardial revascularization, mitral or aortic valve replacement, and endomyocardial biopsies^[16,17]^.

Right CAF to the trunk of the pulmonary artery may be an uncommon cause of syncope in elderly patients^[18]^. More severe complications, such as pulmonary hypertension, congestive heart failure, thrombosis, and arterial aneurysm, may occur due to a large left-right shunt^[19]^. There are two general types of coronary-pulmonary fistula: the first one occurs through a single prominent fistulous connection between the anterior descending artery and the main pulmonary trunk. On the other hand, in the second one, there are multiple fistulous connections between these same arteries. There is a greater symptomatology in patients with a single fistulous connection^[14]^.

## CASE REPORT

A 61-year-old white female patient with a history of dyspnea on moderate exertion since adolescence. About 12 years ago, she also presented precordialgia on moderate efforts, associated with dyspnea, and progressive worsening without electrocardiographic changes at rest. At the time, the patient underwent an ergometric test, which was terminated by precordialgia and fatigue, and coronary angiography was requested. Coronary catheterization revealed atherosclerotic arteries, but three arteriovenous fistulas were present: two major fistulas, originating from the proximal portion of the anterior descending artery, draining into the pulmonary artery, and a third thin fistula, originating from the circumflex artery, also with pulmonary artery drainage.

After a discussion of the *heart team*, percutaneous treatment was indicated, with embolization using coils inside the two fistulas originated from the anterior descending artery, but with an unsatisfactory result and residual flow through both fistulas.

**Fig. 1:**
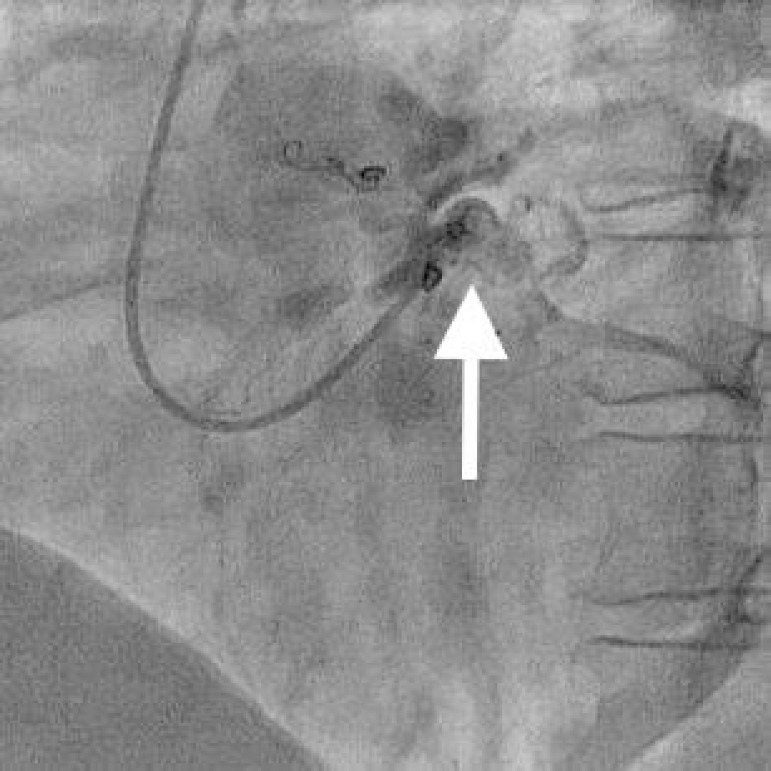
Preoperative coronary angiography demonstrating coronary fistulas with origin from the anterior descending artery and drainage in the trunk of the pulmonary artery

The patient had considerable improvements in symptoms for about eight years. After this period, once again she reported symptoms of precordialgia and dyspnea on moderate exertion. Therefore, a new coronariography was indicated, which demonstrated an increase in the caliber of the three already known fistulas and complete absence of restrictive effect of the coils, with slow flow through the anterior descending artery and large flow sequestration by the fistulas, draining into the pulmonary artery (Figure 1).

This time, a surgical approach was indicated to correct the fistulous pathways, since the patient presented progressive worsening of the symptoms.

**Fig. 2:**
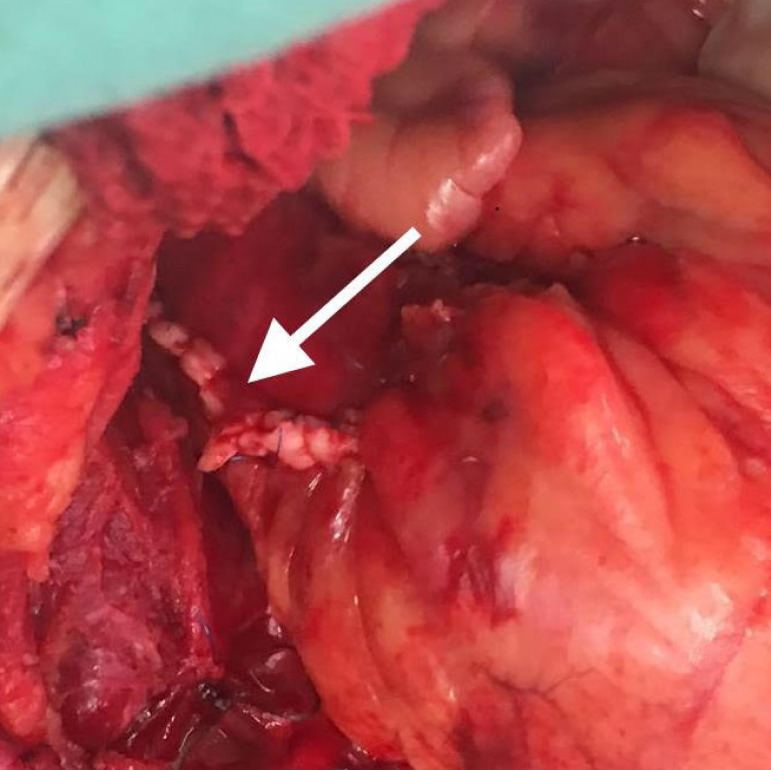
Perioperative aspect of the correction of coronary fistulas and pulmonary artery suture.

On 08/27/2018, coronary fistula correction surgery was performed, by median sternotomy access, using extracorporeal circulation and cardioplegia. We found not only the three fistulas described in coronary angiography, but also a hemangioma involving the root of the pulmonary artery. The fistulas derived from the anterior descending artery, ending in a single drainage ostium in the anterior wall of the pulmonary artery. The three fistulas were properly identified, resected, and occluded with polypropylene monofilament stitches in three segments along their length. The hemangioma approach was performed with the same type of ligature in its major branches and cauterization of the other segments. A longitudinal incision was made in the pulmonary artery, also an identification of the fistulas’ drainage ostia and the ligation of the ostia with polypropylene monofilament stitches (Figure 2).

The postoperative evolution was quite satisfactory, with no need for blood transfusion. The extubation was performed at the 6^th^ hour after the surgery end time. The patient was discharged from the intensive care unit (ICU) on the 2^nd^ postoperative day, leaving the hospital on the 5^th^ postoperative day.

Outpatient follow-up showed complete remission of precordialgia and dyspnea symptoms within the first month after surgical correction, presenting only symptoms related to post-sternotomy healing.

## DISCUSSION

The diagnosis for coronary fistula used to be performed through invasive coronary angiography^[19]^. Recently, the prevalence of coronary angiography has increased, becoming more accurate in detecting coronary fistulas^[20,21]^. Cases of coronary-pulmonary fistulas can be found incidentally because they are asymptomatic, often requiring surgical intervention^[14]^. The case described in this article demonstrates an unusual finding, since the fistulas do not drain directly into the pulmonary artery, but become a complex hemangioma, with common drainage ostium.

The treatment of this type of anomaly is a controversial subject. There are no official clinical guidelines yet, which requires further studies. The surgical approach has positive results even in patients with mitral valve insufficiency and severe left ventricular dysfunction. Regarding hemodynamic repercussions that may occur in these abnormalities, their diagnosis and early treatment are of great importance for medical practice^[3]^.

It has been demonstrated that surgical treatment in patients with coronary-pulmonary fistula is more effective in a follow-up of up to seven years than the percutaneous treatment, presenting lower numbers of mortality and morbidity^[22,23]^. Proper treatment, when used with the support of extracorporeal circulation and myocardial protection, becomes safer for the closure of the fistula ostium. This technique is effective for patients with an ostium of difficult location or a more severe clinical condition^[24]^.

One of the quality markers in the surgical treatment of this type of fistula is the evaluation of the absence of retrograde blood cardioplegia efflux through the intraoperative fistula ostium, since this corroborates to the visualization of the ostium when it has a difficult location^[12]^.

## CONCLUSION

Coronary fistulas have become increasingly frequent with technological advances in hemodynamic studies. Despite a greater demand for further studies proving the superiority of surgical correction over clinical treatment, this case report demonstrated a complete resolution of the patient's symptoms since adolescence, with a substantial improvement in quality of life.

Therefore, we consider surgical treatment as the best therapeutic choice, not only for this case but also for similar situations.

**Table:** 

Authors’ roles & responsibilities	
TCB	Substantial contributions to the conception or design of the work; or the acquisition, analysis, or interpretation of data for the work; final approval of the version to be published.
LS	Substantial contributions to the conception or design of the work; or the acquisition, analysis, or interpretation of data for the work; final approval of the version to be published.
ICO	Substantial contributions to the conception or design of the work; or the acquisition, analysis, or interpretation of data for the work; final approval of the version to be published.
